# *Ligilactobacillus salivarius* CNCM I-4866, a potential probiotic candidate, shows anti-inflammatory properties *in vitro* and *in vivo*

**DOI:** 10.3389/fmicb.2023.1270974

**Published:** 2023-11-29

**Authors:** Celia Carbonne, Sead Chadi, Camille Kropp, Lise Molimard, Florian Chain, Philippe Langella, Rebeca Martin

**Affiliations:** Micalis Institute, AgroParisTech, INRAE, Université Paris-Saclay, Jouy-en-Josas, France

**Keywords:** *Ligilactobacillus salivarius*, probiotic, DNBS inflammation, *in vitro*, anti-inflammatory

## Abstract

**Introduction:**

The aim of this work was to characterize a new strain of *Ligilactobacillus salivarius* (CNCM I-4866) (CNCM I-4866) to address its potential as probiotic with a special focus on intestinal inflammation. Potential anti-inflammatory abilities of this strain were evaluated through *in vivo* and *in vitro* experiments.

**Methods:**

Firstly, the strain was tested in a murine acute inflammation colitis model induced by DNBS. *In vitro* characterization was then performed with diverse tests: modulation capability of intestinal permeability; study of the impact on immunity profile through cytokines dosage; capacity to inhibit pathogens and adhere to intestinal cells lines. Production of metabolites, antibiotic resistance and survival to gastro-intestinal tract conditions were also tested.

**Results:**

*In vitro* assay has shown a reduction of colonic damage and markers of inflammation after treatment with CNCM I-4866. Transcriptomic analysis performed on colons showed the capacity of the strain to down-regulate pro-inflammatory cytokines. *L. salivarius* CNCM I-4866 exerted anti-inflammatory profile by reducing IL-8 production by TNF-α stimulated cell and modulated cytokines profile on peripheral blood mononuclear cells (PBMC). It protected intestinal integrity by increasing trans-epithelial electrical resistance (TEER) on Caco-2 TNF-α inflamed cells. Additionally, *L. salivarius* CNCM I-4866 displayed inhibition capacity on several intestinal pathogens and adhered to eukaryotic cells. Regarding safety and technical concerns, CNCM I-4866 was highly resistant to 0.3% of bile salts and produced mainly L-lactate. Finally, strain genomic characterization allowed us to confirm safety aspect of our strain, with no antibiotic gene resistance found.

**Discussion:**

Taken together, these results indicate that *L. salivarius* CNCM I-4866 could be a good probiotic candidate for intestinal inflammation, especially with its steady anti-inflammatory profile.

## Introduction

Inflammatory bowel diseases (IBDs), including ulcerative colitis and Crohn’s disease, are common chronic gastrointestinal diseases, mainly in Western countries (Aldars-Garcia et al., 2021). IBDs are characterized by inflammation bursting of the gastrointestinal tract driven by over-stimulation of the immune system. This deleterious inflammatory response includes the overproduction of reactive oxygen species, damage to the intestinal epithelial barrier, and an imbalance in the immune response with the secretion of pro-inflammatory cytokines (Jakubczyk et al., 2020).

Even if IBD development is multifactorial, it has been shown that IBDs are linked with deregulation in the intestinal microbiota (Glassner et al., 2020). The intestinal microbiota is composed of more than trillions of different microorganisms, such as bacteria, fungi, or viruses. The majority of intestinal bacteria belong to four phyla: Bacillota, Bacteroidota, Pseudomonadota, and Actinomycetes (Thursby and Juge, 2017). The gut microbiota has many functions, such as host nutrient metabolism, immunomodulation, and protection against pathogens (Jandhyala et al., 2015). In the case of IBD, both the prevalence of pathobionts and/or the lack of key bacteria can promote IBD development and maintenance (Frank et al., 2007).

In the Bacillota phylum, the family Lactobacillaceae is a widely diverse group of Gram-positive bacteria, harboring 25 genera and almost 200 species. They exert an anaerobic metabolism, producing lactic acid through the fermentation of sugars, but are able to tolerate the presence of oxygen. Lactobacilli are widely studied for their potential beneficial properties. For example, some strains are shown to have a beneficial impact on restoring intestinal permeability (Chamignon et al., 2020), which is increased during inflammation. Another key role of some *Lactobacillus* strains is their ability to modulate the immune response in an inflammatory state by decreasing pro-inflammatory cytokine production or enhancing the levels of anti-inflammatory cytokines, such as IL-10 (Alard et al., 2018). Some *Lactobacillus* members are also known to be high producers of exopolysaccharides (EPSs), which have been recognized to directly infer the health-promoting properties of Lactobacilli (Juraskova et al., 2022; Martin et al., 2023).

*Ligilactobacillus salivarius,* formerly named *Lactobacillus salivarius,* is a homo-fermentative species. Many studies have been conducted on *L. salivarius* because of its useful properties for human health (Chaves et al., 2017). Indeed, beyond their capacity to alleviate inflammation-induced colitis in the murine model (Peran et al., 2005; Iyer et al., 2022), some *L. salivarius* strains are well known to exert antimicrobial activity (Tinrat et al., 2011; Messaoudi et al., 2013). Thereby, several strains of the Lactobacillaceae family were characterized these last years as potential probiotics for IBD, serving as complements to treatments in order to reduce disease-associated symptoms. Probiotics are defined as “live microorganisms, that, when administered in adequate amounts, confer a health benefit on the host” (Hill et al., 2014). Due to the wide range of applications of probiotics and their various mechanisms of action, the EFSA has proposed “Guidance on the characterization of microorganisms used as feed additives or as production organisms” (Rychen et al., 2018), suggesting safety and effectiveness criteria for their evaluation. These criteria include mostly physiological characterization and safety assessment by genomic analysis, particularly research for antibiotic resistance. Other key features to ensure the strain’s ability to exert its beneficial activity are resistance to the gastrointestinal tract or adhesion to the intestinal mucosa.

In this study, we have assessed the probiotic capacities of *L. salivarius* CNCM I-4866 to target gut inflammation as the first step to characterize its potential beneficial effects on IBD management. Murine acute colitis assays and *in vitro* experiments were conducted to target some potentially beneficial effects observed *in vivo* (restoration of intestinal permeability and immunomodulatory response). Technological and safety parameters and genomic characterization were also explored to validate the safe status of this strain and its industrial interest.

## Materials and methods

### Growth of strains and eukaryotic cells

*Ligilactobacillus salivarius* CNCM I-4866 was isolated by SORBIAL company from the rumen of grazing lamb. *Ligilactobacillus salivarius* CNCM I-4866 and *Lacticaseibacillus rhamnosus* GG were grown in Man Rogosa and Sharpe (MRS) (Biokar, Solabia, France) at 37°C in aerobic conditions.

Bacterial cultures were centrifuged at 8000xg, washed twice in DPBS (Gibco, Thermo Fisher, USA), and resuspended in DPBS at an established concentration. These aliquots were used for *in vitro* and *in vivo* assays described below.

The human colon adenocarcinoma cell lines HT-29 and Caco-2 were obtained from the American Type Culture Collection (ATCC, United Kingdom). HT-29 cells were grown in Dulbecco’s modified Eagle’s medium supplemented with GlutaMAX (DMEM) (Gibco, Thermo Fisher) and 10% (v/v) of heat-inactivated fetal bovine serum (FBS, Eurobio, France). The Caco-2 cell line was cultured on DMEM GlutaMAX medium, 10% FBS, 1% non-essential amino acids (Gibco, Thermo Fisher), and 0.1% penicillin/streptomycin (Gibco, Thermo Fisher). The cultures were maintained at 37°C under 10% CO_2_, and the medium was changed every 2 days.

### DNBS-induced colitis assays

**Figure 1:**
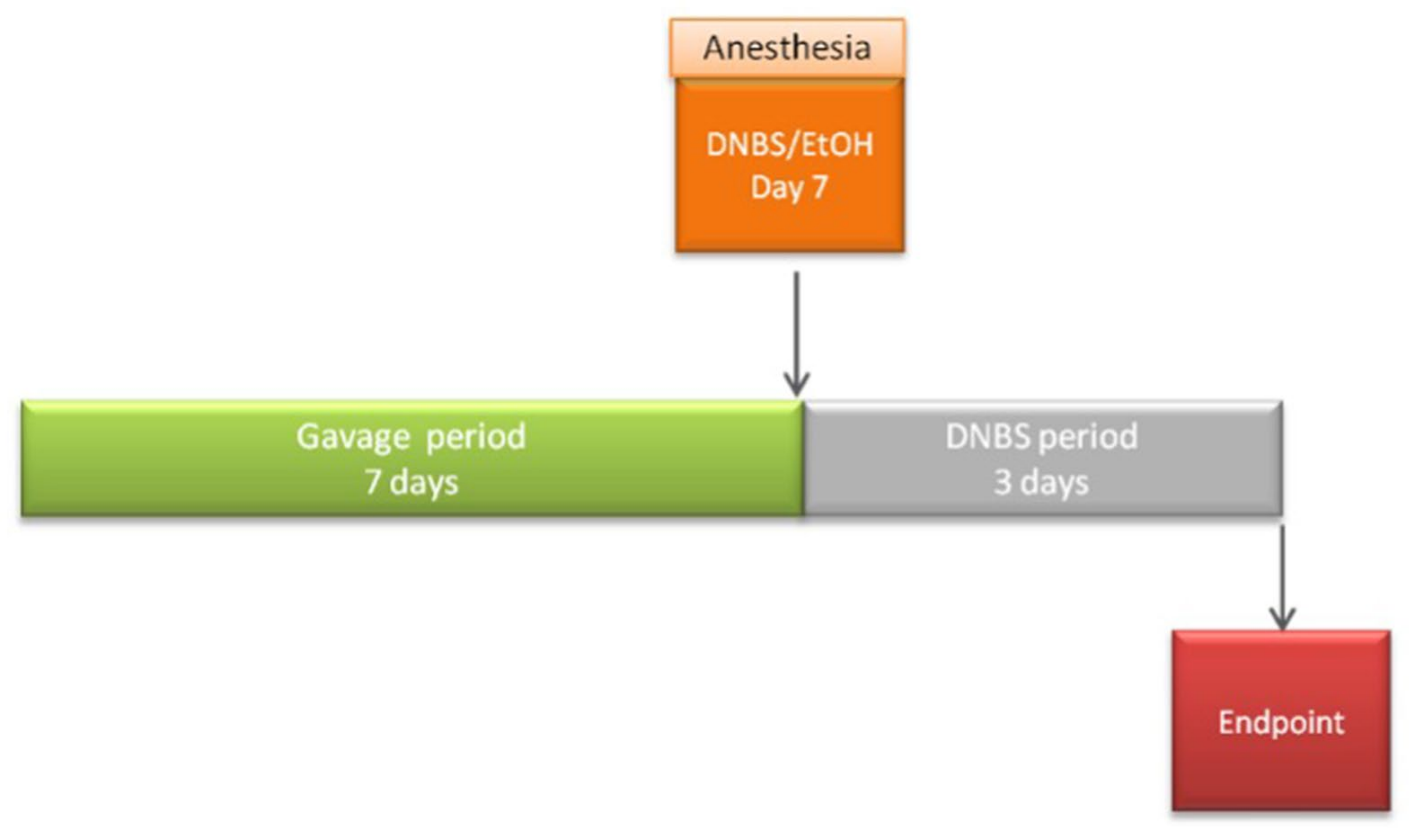
Procedure description of the acute DNBS model.

For DNBS assays, 54 6-week-old male C57BL/6JrJ mice were obtained from Janvier Laboratory (Janvier, France) and maintained under specific pathogen-free (SPF) conditions in the animal facilities of the IERP Experimental Unit, INRAE. They were housed in four or five cages. Experiments were performed in accordance with European Union legislation on animal welfare and were approved by COMETHEA, a local committee on animal experimentation (no. 16744–201807061805486), and in compliance with the ARRIVE relevant guidelines. After a 7-day acclimation period, 54 mice were divided into 3 groups (*n* = 8 or *n* = 10 mice/group): the vehicle control group [no inflammation; Ethanol (EtOH)-Vehicle], the inflamed control group (inflammation-induced; DNBS-Vehicle), and the treated group (DNBS-CNCM I-4866). For 10 days, intra-gastric administration of DPBS (Vehicle) (Gibco, Thermo Fisher) (200 μL), 16% (v/v) glycerol, or the bacteria resuspended in DPBS (10^9^ CFU/mL in 200 μL) was performed. Gavages were performed daily until the end of the experiment. After 7 days, the mice were anesthetized with an intraperitoneal injection of 0.1% ketamine and 0.06% xylazine. Subsequently, an intra-rectal injection of 2,4-dinitrobenzenesulfonic acid hydrate (Sigma, Switzerland) at a 2.75 mg/mice concentration dissolved in 30% ethanol in DPBS was injected. The vehicle control group received an intra-rectal injection of 30% ethanol in DPBS alone. After 3 days of the injection, blood was collected from the sub-mandibular vein, and mice were euthanized by cervical dislocation. The experiment procedure is presented in Figure 1.

Macroscopic scores in terms of Wallace scores (Wallace et al., 1989), microscopic scores in terms of Ameho scores (Ameho et al., 1997), and myeloperoxidase (MPO) activity levels were determined on the colon samples as described before (Barone et al., 2018). The levels of lipocalin-2 (Mouse Lipocalin-2, R&D Systems, USA) and sCD14 (Mouse sCD14, R&D Systems, USA) were determined using ELISA, according to the manufacturer’s instructions.

Transcriptomic analysis was performed on the colon samples of mice. In brief, samples were conserved in RNA at −80°C, and RNA was extracted afterward with the RNeasy kit (RNeasy kit, Qiagen, the Netherlands), following the manufacturer’s instructions. Transcriptomic analysis was performed by the GENOM’IC platform at Cochin Institute using the 3’tag method. FASTQ files were then aligned using the STAR algorithm (version 2.7.6a) on the Ensembl release 101 reference. Reads were then counted using RSEM (v1.3.1), and the statistical analyses on the read counts were performed with R (version 3.6.3) and the DESeq2 package (DESeq2_1.26.0) to determine the proportion of differentially expressed genes between two conditions. The standard DESeq2 normalization method (DESeq2’s median of ratios with the DESeq function) was used, with pre-filter of reads and genes (reads uniquely mapped on the genome, or up to 10 different loci with a count adjustment, and genes with at least 10 reads in at least 3 different samples). Following the package recommendations, the Wald test with the contrast function and the Benjamin–Hochberg FDR control procedure were used to identify the differentially expressed genes. Selected gene lists (|log2FoldChange| > 1.5 and value of *p* < 0.05) were loaded into ingenuity pathway analysis (IPA) to analyze pathways and generate data.

### Anti-inflammatory *in vitro* assay on HT-29 cells

HT-29 cells were seeded into 24-well plates (1 × 10^5^ cells per well). After 6 days, when confluence was reached, the medium was replaced by a DMEM GlutaMAX medium with 5% FBS. After 24 h, on day 7, co-incubation with the bacterial cells was performed at a multiplicity of infection (MOI) of 40 in DMEM GlutaMAX, 0.1% penicillin/streptomycin, and 5% FBS and supplemented or not with TNF-α at a final concentration of 5 ng/mL (PeproTech, USA). DPBS was used as a negative control and butyrate at 10 mM as a positive control. After 6 h of co-incubation, supernatants were recovered and stored at −80°C. Interleukin (IL)-8 concentrations were quantified using the Human IL-8 ELISA MAX Standard Set (BioLegend, USA), according to the manufacturer’s instructions. The absorbance was measured at 450 nm using the Infinite M200 Pro (TECAN, Switzerland).

### Transepithelial resistance measurements

Caco-2 cells were grown on Transwell inserts and kept at 37°C under 10% CO_2_ until 80% confluence was reached. The medium was changed every 2 days. When optimal transepithelial resistance (TEER) values were reached (REMS AutoSampler, World Precision Instruments, USA), the fresh medium was added. Then, the strain *L. salivarius* CNCM I-4866 and *Lacticaseibacillus rhamnosus* GG (used as a positive control; Chamignon et al., 2020) at MOI 40 or the control (DPBS) were added to the apical compartment of the cells. After 3 h, 100 ng/mL of TNF-α was added to the basal compartment of Transwell plates. TEER was measured just before and 24 h after the treatments. The results were normalized to basal TEER as follows:


$$\mathrm{Ratio}=\frac{\mathrm{TEERTreatmentT}24/\mathrm{TEERTreatmentT}0}{\mathrm{TEERControlT}24/\mathrm{TEERControlT}0}$$

### Immunomodulatory effects on PBMCs

Human peripheral blood mononuclear cells (PBMCs) isolated from the blood of healthy donors were obtained from StemCells (StemCells, Canada) and stored in liquid nitrogen. Five donors were selected according to the next selective criteria as follows: male individuals, BMI between 20 and 30, non-smokers, and with no allergies or diseases, such as asthma. After thawing, PBMCs were washed twice with Roswell Park Memorial Institute GlutaMAX medium (RPMI) (Gibco, Thermo Fisher) containing 10% FBS, and DNase I was added to avoid aggregate formation. Then, cells were centrifuged at 200 *g* for 15 min at room temperature, and the supernatants were discarded. The washing step was performed twice, and then PBMCs were counted using the trypan blue method. Next, PBMCs were seeded at a rate of 1 million per 24-well plate. In total, 50 μL of fresh bacteria cultured in MRS (Difco, Thermo Fisher) was added at an MOI of 10, and co-cultures were maintained at 37°C in 5% CO_2_ for 24 h. *Escherichia coli* TG1 was used as a control (Sokol et al., 2008). Finally, supernatants were collected, and interleukin-10 (IL-10), interleukin-12 (IL-12), and tumor necrosis factor (TNF-α) were quantified by ELISA using specific kits (Mabtech, Sweden), according to the manufacturer’s guidelines.

### Ability to inhibit pathogen growth

Pathogen inhibition capacity against eight pathogens was determined. *Salmonella typhimurium, Salmonella enteritidis, Listeria monocytogenes* EDGE, *Escherichia coli* ATCC 700928, *Staphylococcus aureus* CNRZ 875, and *Clostridium perfringens* ATCC 13124 were obtained from the INRAE internal collection. *Helicobacter pylori* 26,695 was kindly provided by the Pasteur Institute (Dr. Hilde de Reuse team). *Campylobacter jejuni* BF was provided by the INRAE/ONIRIS Nantes collection. The first six strains were cultivated on Mueller Hinton (Thermo Fisher) at 37°C under aerobic conditions. *H. pylori* and *C. jejuni* were cultivated on Mueller Hinton agar supplemented with 5% sheep blood (Thermo Fisher) at 37°C in a micro-aerophilic atmosphere (bioMérieux, France). To perform inhibition tests, a lawn of each pathogen from a fresh suspension was made on Mueller Hinton. Holes were made in the agar with P100 sterile tips, to which 50 μL of filtered supernatants from a stationary-phase culture or control medium alone (MRS) was added. The results were read after 48 h of incubation as the diameter of inhibition (mm). To assess whether inhibition was due to acid production by the *L. salivarius* CNCM I-4866 strain, we performed the assay with supernatants neutralized at pH 7 with sodium hydroxide.

### Adhesion capacity tests

To assess bacterial adhesion capacity, Caco-2, HT-29, and its derivative, HT-29 MTX, were used. Cells were seeded into a 24-well tissue culture plate at a concentration of 1 × 10^5^ cells/well, and adhesion was performed for 7 days for Caco-2 and HT-29 ATCC. For HT-29 MTX, after confluence (6 days), plates were incubated for an additional 14 days to allow cell differentiation (the medium was changed every day). In all cases, after 7 days or 21 days, wells were washed twice with DPBS, and fresh media without antibiotics were added. Each bacterial suspension was added at MOI 40 from a stationary-phase culture. After 3 h of incubation, monolayers were washed three times with DPBS to remove any bacteria that were not attached to the cells. Afterward, bacteria were disassociated by covering the monolayer with 150 μL of a 1% (v/v) Triton (Triton X-100, Sigma) solution in DPBS. Subsequently, 300 μL of DMEM was added in order to stop the reaction, and the number of viable adherent bacteria was determined by plating serial dilutions on MRS agar plates. Adhesion was expressed as the percentage of adhered bacteria with respect to the number of input bacteria, or DPBS as a negative control.

The adhesion test was also performed on mucin (Porcine gastric mucin, type III, Sigma). Mucin was prepared at 10 mg/mL in sterile DPBS and put on a 96-well plate overnight at 4°C. Adhesion assay was performed as described above, with an additional incubation at room temperature for 90 min after adding Triton solution.

### Determination of D-,L-lactate concentrations

D-lactate and L-lactate were measured in the supernatant of the bacterial culture at the stationary phase. The supernatant was precipitated with trichloroacetic acid (10%) and centrifuged at 20,000 *g* for 5 min at 4°C. Acid supernatants were neutralized with TEA 0.1 M at pH 9.15. Lactate was then measured with an enzymatic kit according to the manufacturer’s instructions (Biosentec, France).

### Antibiotic resistance determinations

Phenotypic resistance to antibiotics was assessed according to the EFSA recommendations (European Food Safety Authority, 2018). Lactic acid bacteria susceptibility test medium (LSM agar) was prepared with 90% IST (Iso-Sensitest broth, Oxoid, United Kingdom), 10% MRS broth, and 1.5% granulated agar. Bacterial suspensions were streaked on plates to obtain a lawn, and antibiotic strips (bioMérieux) were used. The inhibition area with the corresponding concentration (minimum inhibitory concentration, MIC) was then determined and compared with the EFSA guidelines.

### Genomic characterization

Genomic DNA was extracted from 5 mL of culture with the first step of enzymatic lysis with the following cocktail: mutanolysin at 233.3 U/mL; lysostaphin at 13.3 U/mL, and lysozyme at 50 mg/mL, followed by incubation with RNAse A (Qiagen) at 10 mg/mL and proteinase K (Euromedex, France) at 50 mg/mL. Purification was performed with a DNA extraction kit (Genomic DNA Buffer Set and Genomic Tips, Qiagen), according to the manufacturer’s instructions. DNA was resuspended in TE buffer, and the concentration was measured with NanoDrop (NanoDrop 1,000, Thermo Fisher). The genome was sequenced by Eurofins Genomics (France) using whole-genome sequencing with *de novo* assembly, with the PacBio method on single-molecule real-time (SMRT) cells, a 240-mn collection time, a mean length superior to 6,000 bp, and a genome coverage of 100 X. The analysis of the obtained reads began with a quality check and *de novo* assembly of contigs. Contigs were then circularized and mapped when possible.

The genome is available at NCBI (BioSample accession ID: SAMN37542358). The presence of antibiotic gene resistance was searched online on two databases, namely, CARD^1^ and ResFinder.^2^ According to the “EFSA statement on the requirements for whole genome sequence analysis of microorganisms intentionally used in the food chain” (European Food Safety Authority, 2021), only hits with 80% identity and 70% length were reported. The presence of prophages in the genomes was determined *in silico* using Phaster.^3^ Only intact prophages were considered. Potential bacteriocin activity was determined *in silico* using BAGEL4.^4^

### Bacteriophage induction essays

An induction assay was performed to establish if the intact prophage found with Phaster was active. In brief, induction with mitomycin C (Sigma) at 1 μg/mL was performed on culture at the beginning of the exponential phase. When the culture reached the stationary phase, 1 mL of culture was centrifuged (8,000 *g*, 10 mn, 4°C), and the supernatant was then filtered (0.22 μM) before being frozen at −20°C*. Listeria ivanovii* WSCL 3009 (Institute for Food and Health from the Technical University of Munich, Germany) was used as a receptor strain for B025 prophage. Two different protocols were performed with MRS agar (1.5%) and semi-solid MRS agar (0.75%), supplemented with 2 mM of CaCl_2_ to increase phage absorption. On spot assay, 100 μL of receptor bacteria at the beginning of the exponential phase culture was poured with semi-solid agar on top of solid agar. In total, 10 μL of inducted supernatant or control was spotted on the surface of the Petri dish. On double lawn assay, 100 μL of receptor bacteria at the beginning of exponential phase culture was mixed with 100 μL of inducted supernatant or control. After 15 min of incubation at room temperature, 3 mL of semi-solid agar was added and then poured on solid agar. After 48 h of incubation, the presence of potential inhibition halos, indicating the presence of active phages, was observed.

### Active bacteriocin determination test

Potential bacteriocin activity was determined using a sensitive strain to enterolysin (which is the bacteriocin predicted for our strain): *Lactococcus lactis* IL403. The bacterial suspension was collected from the fresh colony of sensitive bacteria in sterile peptone water (McFarland 1) and streaked on LSM medium (medium prepared as described before). In total, 10 μL of supernatant was added to the agar. Three conditions were tested for each supernatant: filtered only; filtered and pH adjusted to 6; and filtered with pH 6 and 1 mg/mL of catalase, which inhibits potential H_2_O_2_ action. A bacterial growth medium was used as a negative control. Potential inhibition spots were observed after 48 h of incubation.

### Hemolytic activity

The hemolytic activity of our strain was determined by using blood agar, which was streaked with our strain and incubated at 37°C for 48 h. After incubation, the hemolytic activity was evaluated and classified based on the lysis of red blood cells in the medium around the colonies.

### Bile salts and pH resistance

Tolerance to bile salts was studied to mimic the passage of the strains in the gastrointestinal tract. From stationary-phase culture, bacterial cells were exposed to 0% or 0.3% bile salts (Oxgall Powder, Sigma) for 1 h before viable cells were counted. Second, viable counts were performed after 24 h of growth of the *L. salivarius* CNCM I-4866 strain in media containing 0% or 0.3% bile salts.

Tolerance to acidic conditions was tested in the same conditions for bile salts by following the growth in media with a modified pH or after 1 h of exposition to a low pH and counting viable cells. We have performed assays at pH 2 and pH 4.

### Exopolysaccharide production: Ropy test and transmission electron microscopy

The production of potential exopolysaccharides (EPSs) by *L. salivarius* CNCM I-4866 was quantified with the Ropy phenotype test. In brief, a loop was used to observe EPS filament from a fresh colony on agar.

To determine bacterial structures, transmission electron microscopy (TEM) was performed by the microscopy and imaging platform (MIMA2, INRAE). The bacterial pellet obtained from an exponential phase was washed twice with phosphate buffer and recovered with 2% glutaraldehyde (EMS) in sodium cacodylate 0.1 M buffer. Suspensions were incubated for 1 h at room temperature. The pellet was then washed with sodium cacodylate in 0.1 M buffer (Fluka) and supplemented with 0.2 M sucrose. The sample was conserved at 4°C before being processed. The Hitachi HT7700 (Hitachi High-Tech, Japan) was used for microscopic observations.

## Results

### *Ligilactobacillus salivarius* CNCM I-4866 protects against DNBS-induced colitis inflammation

**Figure 2:**
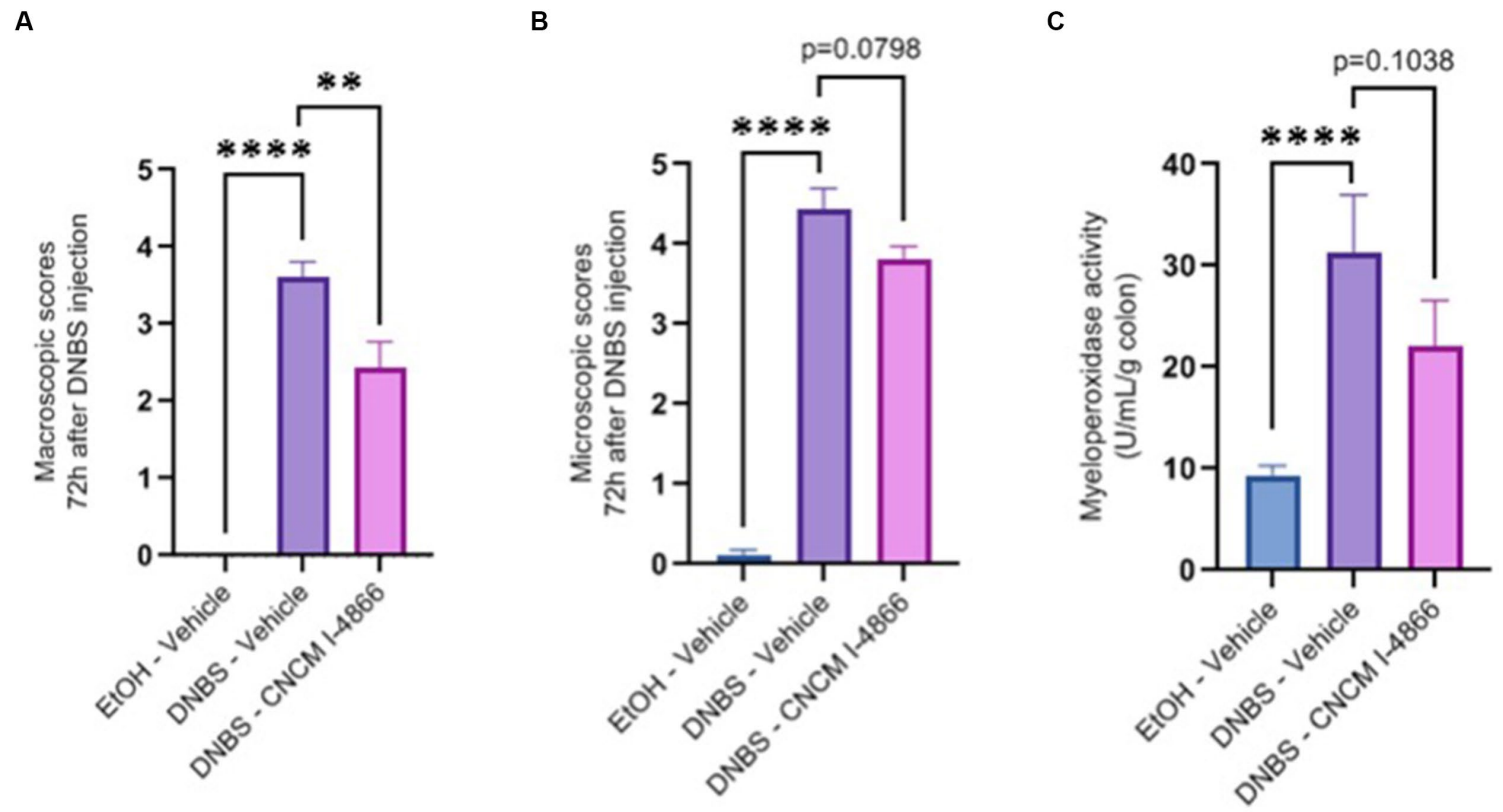
Effects of *L. salivarius* CNCM I-4866 on DNBS-induced colitis. **(A)** Colon macroscopic scores (Wallace scores); **(B)** colon microscopic scores (Ameho scores); and **(C)** levels of MPO activity in the colon. Results of Mann–Whitney *U*-tests compared with the DNBS-Vehicle group with the EtOH-Vehicle and treated groups: *p < 0.05, **p < 0.01, ***p < 0.001, and ****p < 0.0001.

DNBS-induced colitis was performed to observe a potential protective effect of the strain on acute inflammation. A significant decrease was observed in the macroscopic scores of the treated group compared with the DNBS-Vehicle group, indicating lower inflammation in the treated group (Figure 2A). Microscopic scores and myeloperoxidase (MPO) activity determinations (Figures 2B,C) showed a tendency to recovery (with *p*-values of 0.0798 and 0.1038), reflecting an improvement in colonic epithelial structure and reduced immune cell infiltration.

**Figure 3:**
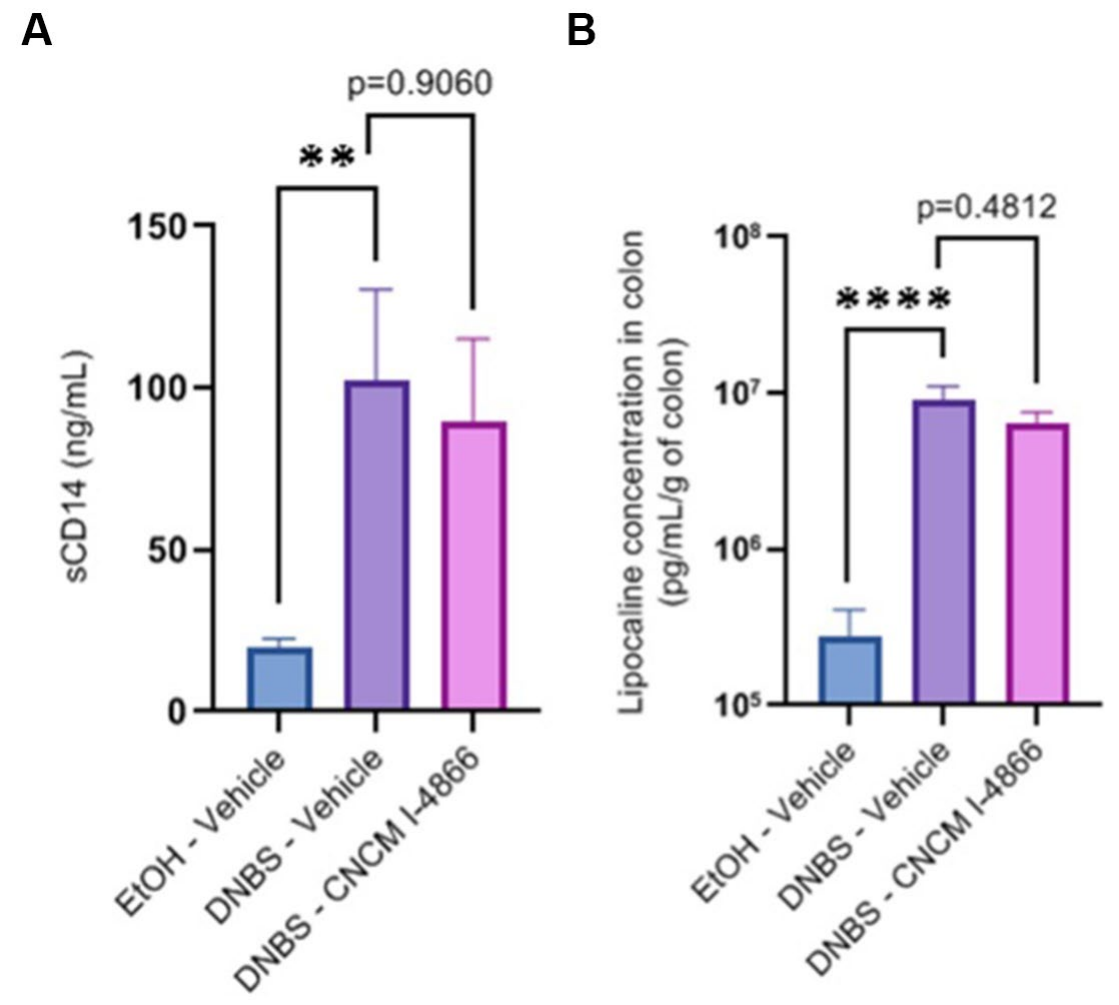
Effects of the *L. salivarius* 4,866 strain on inflammation markers in DNBS-induced colitis. **(A)** Levels of sCD14 in serum and **(B)** levels of lipocalin in the colon. Results of Mann–Whitney *U*-tests compared with the DNBS-Vehicle group with the EtOH-Vehicle and treated groups: *p < 0.05, **p < 0.01, ***p < 0.001, and ****p < 0.0001.

Intestinal permeability is known to increase in cases of inflammation (Salvo-Romero et al., 2015). *Ligilactobacillus salivarius* I-4866 showed a tendency to decrease sCD14, an indicator of permeability, compared with the DNBS-Vehicle group (Figure 3A). Moreover, *L. salivarius* CNCM I-4866 treatment tended to decrease LCN-2 concentration in serum (Figure 3B), a biological marker of inflammation.

### Transcriptome analysis reveals that *Ligilactobacillus salivarius* 4,866 downregulates pro-inflammatory cytokine pathways

**Figure 4:**
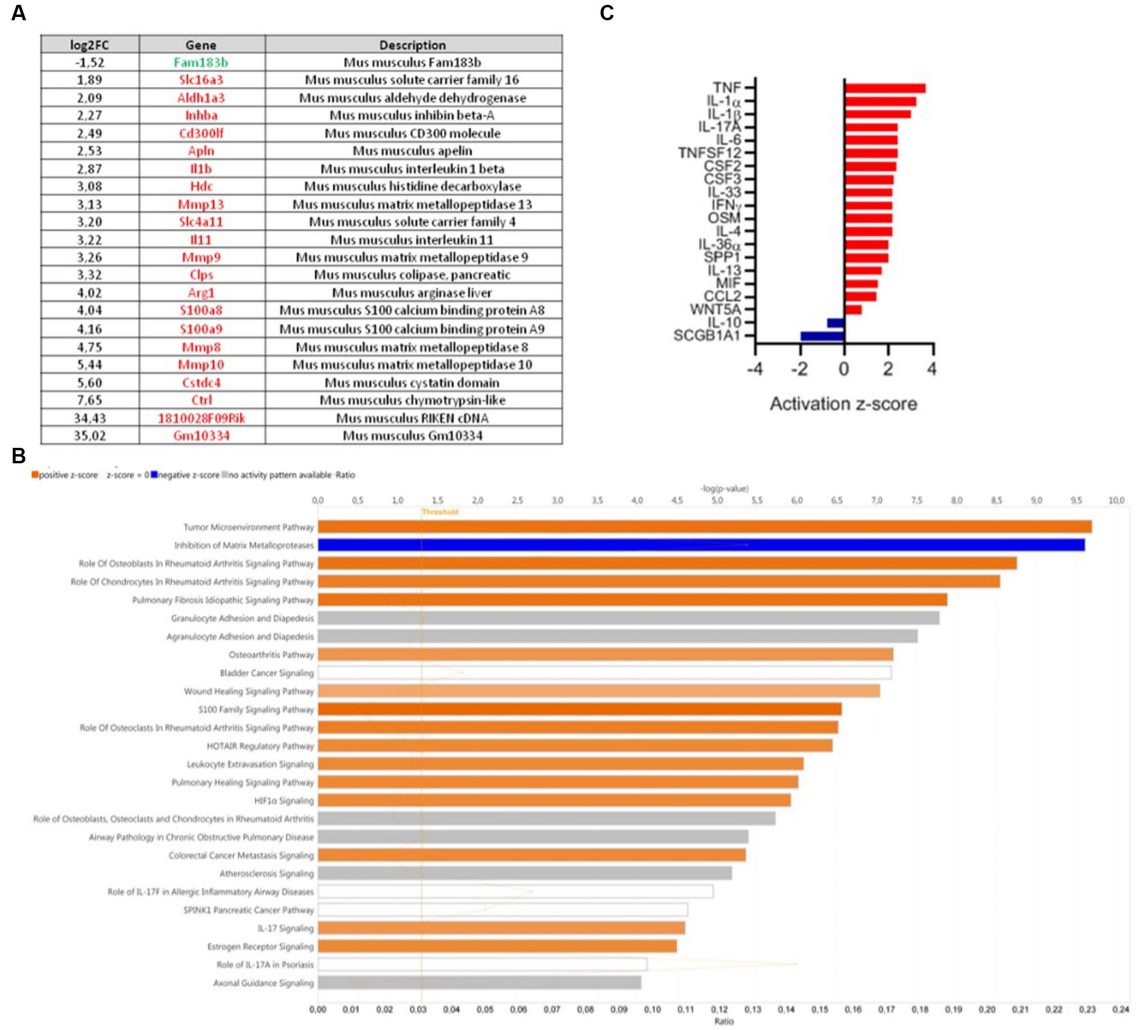
Transcriptomic analysis of mouse colons from the DNBS-Vehicle or DNBS-CNCM I-4866-treated group. **(A)** Modulation of genes between the DNBS-Vehicle group and DNBS-CNCM I-4866-treated group (adjusted value of *p* < 0.05 and |log2FoldChange| > 1.5). Upregulated genes are in red and downregulated genes in green; **(B)** IPA canonical pathway display of the genes modulated in a comparison of DNBS-Vehicle versus DNBS-CNCM I-4866: the *y*-axis displays the -log of the value of *p*, which is calculated by a right-tailed Fisher’s exact test. The orange- and blue-colored bars indicate predicted pathway activation or predicted inhibition, respectively. The orange points interconnected by a thin line represent the ratio; **(C)** Top 20 of affected upstream regulators (only cytokines are represented here) based on IPA. Red indicates activation, while blue indicates suppression.

Comparative colonic transcriptomic analysis revealed that 22 genes were modulated between the DNBS-CNCM I-4866 and DNBS-Vehicle groups (Figure 4A). Among them, inflammatory cytokines were found. IL-1β was one of the genes linked to inflammation that was less expressed in the CNCM I-4866-treated group. Analysis of the specific signaling pathways modulated between these two groups (Figure 4B) also revealed that the tumor environment pathway is the top activated pathway in DNBS-Vehicle (Figure 4B). In addition, several pro-inflammatory cytokines (IL-1α, TNF, IL-1β, and IFNγ, among others) were also found as upstream regulators with significant *z*-scores (Figure 4C) when comparing the DNBS-Vehicle and DNBS-CNCM I-4866 groups. In other words, these results indicated that CNCM I-4866 treatment moderated the production of pro-inflammatory cytokines, as mentioned above. On the other hand, IL-10, an anti-inflammatory cytokine, had a negative *z*-score, indicating that it was downregulated in the DNBS-Vehicle group compared with the treated group. These results suggest that IL-10 could be more expressed in the colon of mice that have received CNCM I-4866.

### *Ligilactobacillus salivarius* 4866 displayed anti-inflammatory capabilities and restored intestinal permeability *in vitro*

**Figure 5:**
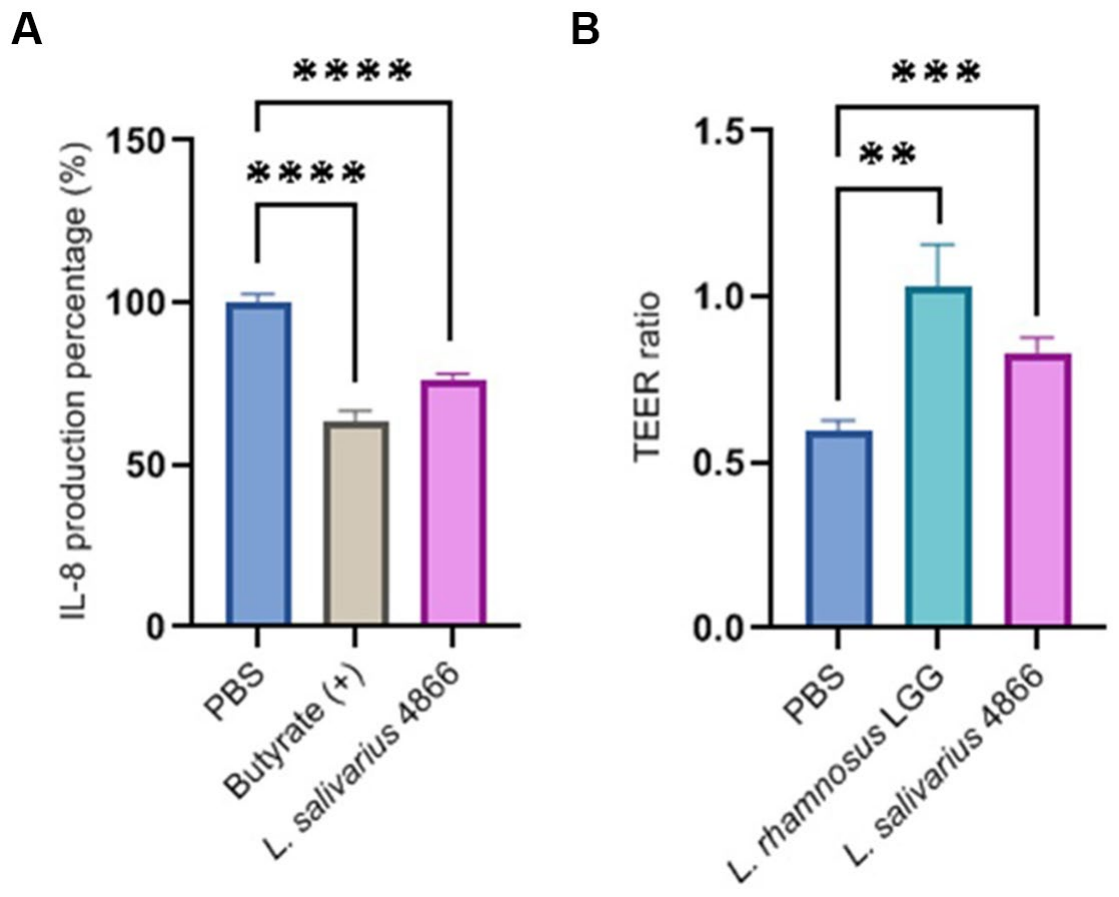
*In vitro* beneficial capacities of the *L. salivarius* CNCM I-4866 strain. **(A)** IL-8 production by HT-29 cells exposed to TNF-α in the presence of *L. salivarius* CNCM I-4866 and butyrate (positive control); **(B)** TEER ratio of Caco-2 cells monolayer exposed to TNF-α in the presence of *L. salivarius* CNCM I-4866 and *L. rhamnosus* GG. The results of Mann–Whitney *U*-tests comparing the DPBS control group with the other groups: *p < 0.05, **p < 0.01, ***p < 0.001, and ****p < 0.0001.

The capacity of *L. salivarius* CNCM I-4866 to modulate TNF-α-induced secretion of IL-8, a major pro-inflammatory cytokine, and limit inflammation was conducted on HT-29 cells. In a similar way to the positive control butyrate (Lenoir et al., 2020), our strain has shown great ability to reduce IL-8 production on inflamed HT-29 cells (Figure 5A).

TEER results have shown that *L. salivarius* CNCM I-4866 treatment was able to maintain barrier integrity in Caco-2 cells challenged with TNF-α with a slight lower effect that the well-known probiotic candidate *L. rhamnosus* GG (Figure 5B).

**Figure 6:**
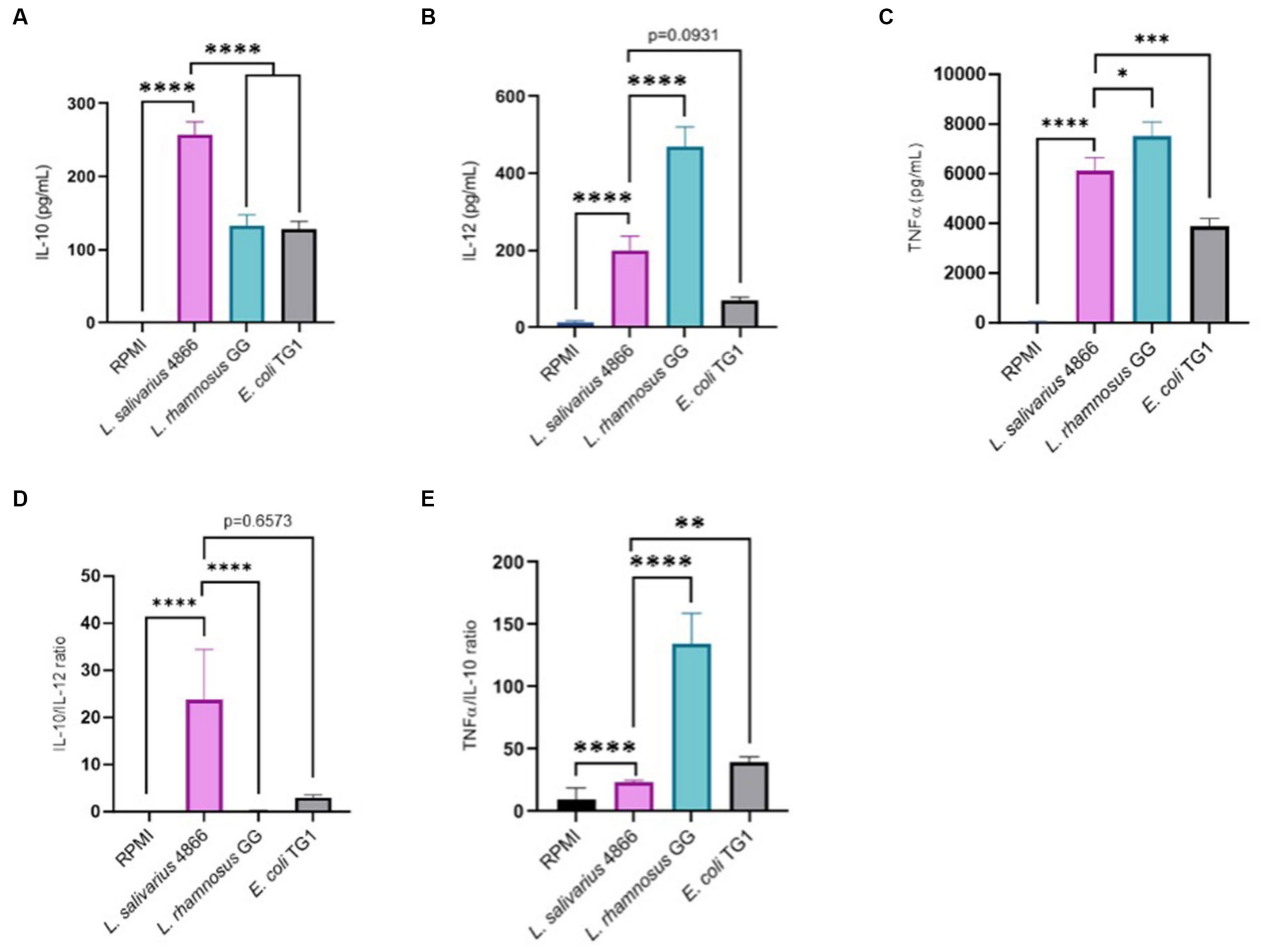
Anti-inflammatory profile based on co-incubation with human PBMCs. **(A)** IL-10 production by *L. salivarius* CNCM I-4866 strain after co-incubation with human PBMCs for five donors. *L. rhamnosus* GG and *E. coli* TG1 are used as controls with known effects; **(B)** IL-12 production by *L. salivarius* CNCM I-4866 strain after co-incubation with human PBMCs, for 5 donors. *L. rhamnosus* GG and *E. coli* TG1 are used as controls with known effects; **(C)** TNF-α production by *L. salivarius* CNCM I-4866 strain after co-incubation with human PBMCs, for 5 donors. *L. rhamnosus* GG and *E. coli* TG1 are used as controls with known effects; **(D)** IL-10/IL-12 ratio for *L. salivarius* CNCM I-4866 after co-incubation with human PBMCs. A low ratio is a marker of the pro-inflammatory profile, whereas a high ratio is a marker of the anti-inflammatory profile; **(E)** TNFα/IL-10 ratio for *L. salivarius* CNCM I-4866 after co-incubation with human PBMCs. A low ratio is a marker of an anti-inflammatory profile, whereas a high ratio is a marker of a pro-inflammatory profile. Results of Mann–Whitney U-tests comparing *L. salivarius* 4,866 with other groups: *p < 0.05, **p < 0.01, ***p < 0.001, and ****p < 0.0001.

Immunomodulatory effect analysis was also conducted on PBMC from five human donors with the same criteria. Pro-inflammatory cytokines (IL-12) and anti-inflammatory cytokines (IL-10) were dosed after co-incubation of PBMC cells with *L. salivarius* CNCM I-4866, *L. rhamnosus* GG, and the control *E. coli* TG1 (Figures 6A,B). *E. coli* TG1 is known to produce IL-10 and not IL-12, thus having an elevated IL-10/IL-12 ratio (Sokol et al., 2008). *Ligilactobacillus salivarius* CNCM I-4866 showed an anti-inflammatory profile based on its high production of IL-10 and high ratio of IL-10/IL-12 (Figure 6D). Moreover, TNF-α production was measured (Figure 6C) in order to determine the TNF-α /IL-10 ratio, which is also an indicator of the inflammatory profile. It appeared to be relatively low for *L. salivarius* CNCM I-4866 compared with *L. rhamnosus* GG and equivalent to the *E. coli* TG1 ratio (Figure 6E).

### *Ligilactobacillus salivarius* CNCM I-4866 had good adhesion capacities and was able to inhibit several pathogens due to its acid production

**Figure 7:**
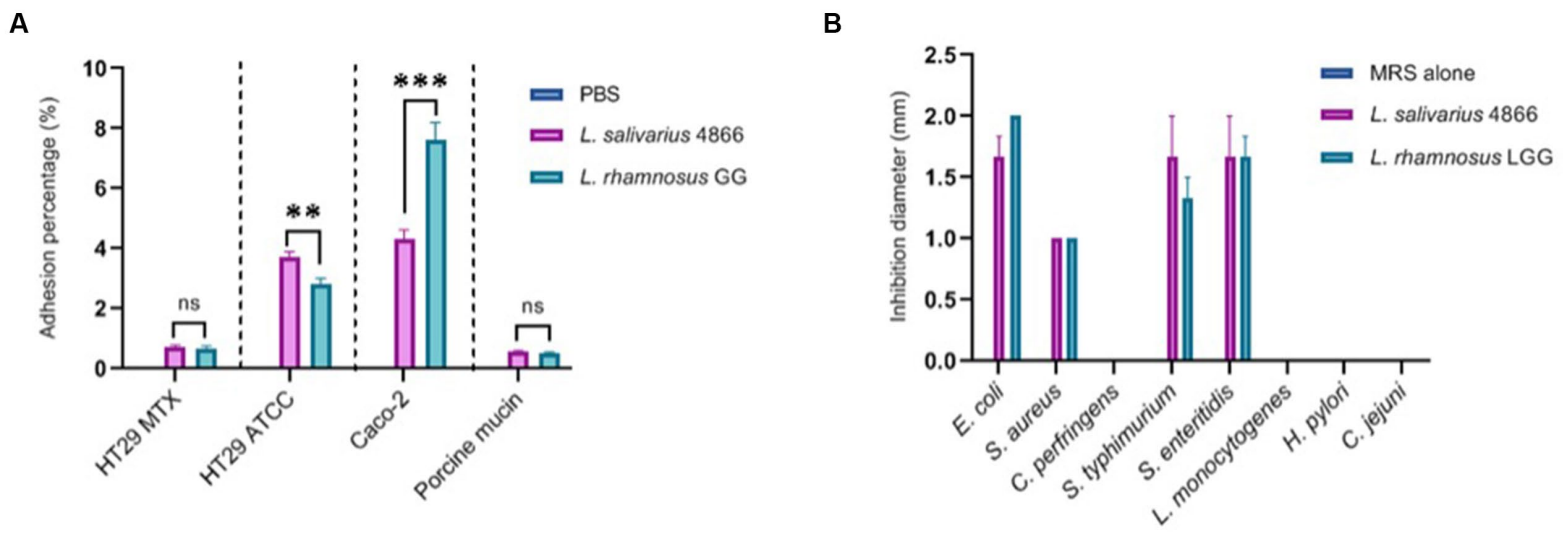
*Ligilactobacillus salivarius* CNCM I-4866 adhesion and pathogen inhibition *in vitro* **(A)** Adhesion percentage to HT-29 MTX, HT-29, Caco-2 cells, and to porcine mucin. Percentage is the ratio of viable count (CFU/ml) after co-incubation compared with the initial inoculum. *L. rhamnosus* GG was used as a positive control; **(B)** pathogen inhibition ability of eight pathogens in the *L. salivarius* CNCM I-4866 supernatant. *L. rhamnosus* GG was used as a positive control. Results of Mann–Whitney U-tests comparing *L. salivarius* 4866 and *L. rhamnosus* GG: **p < 0.01, ***p < 0.001.

We have studied *L. salivarius* CNCM I-4866 ability to adhere to several cell lines: HT-29, HT-29 MTX, and Caco-2, as well as porcine mucin (Figure 7A). Comparatively to the reference strain *L. rhamnosus* GG, which is known to have a good adhesion capacity (Tuomola and Salminen, 1998; Ayeni et al., 2011), *L. salivarius* CNCM I-4866 has shown a good capacity to adhere to HT-29 and Caco-2 cells. Adhesion to HT-29 MTX cells and porcine mucin was more moderate but similar to *L. rhamnosus* GG control.

The ability of the strain supernatant to inhibit eight pathogens was also tested. *Ligilactobacillus salivarius* CNCM I-4866 supernatant inhibited four strains: *Escherichia coli, Staphylococcus aureus, Salmonella typhimurium,* and *S. enteritidis* (Figure 7B). This inhibition effect was lost when the pH of the supernatant was increased by adding sodium hydroxide, which indicates that the inhibition effect was probably due to lactic acid production.

### *Ligilactobacillus salivarius* CNCM I-4866 resisted bile salt exposition and showed no hemolytic activity

**Figure 8:**
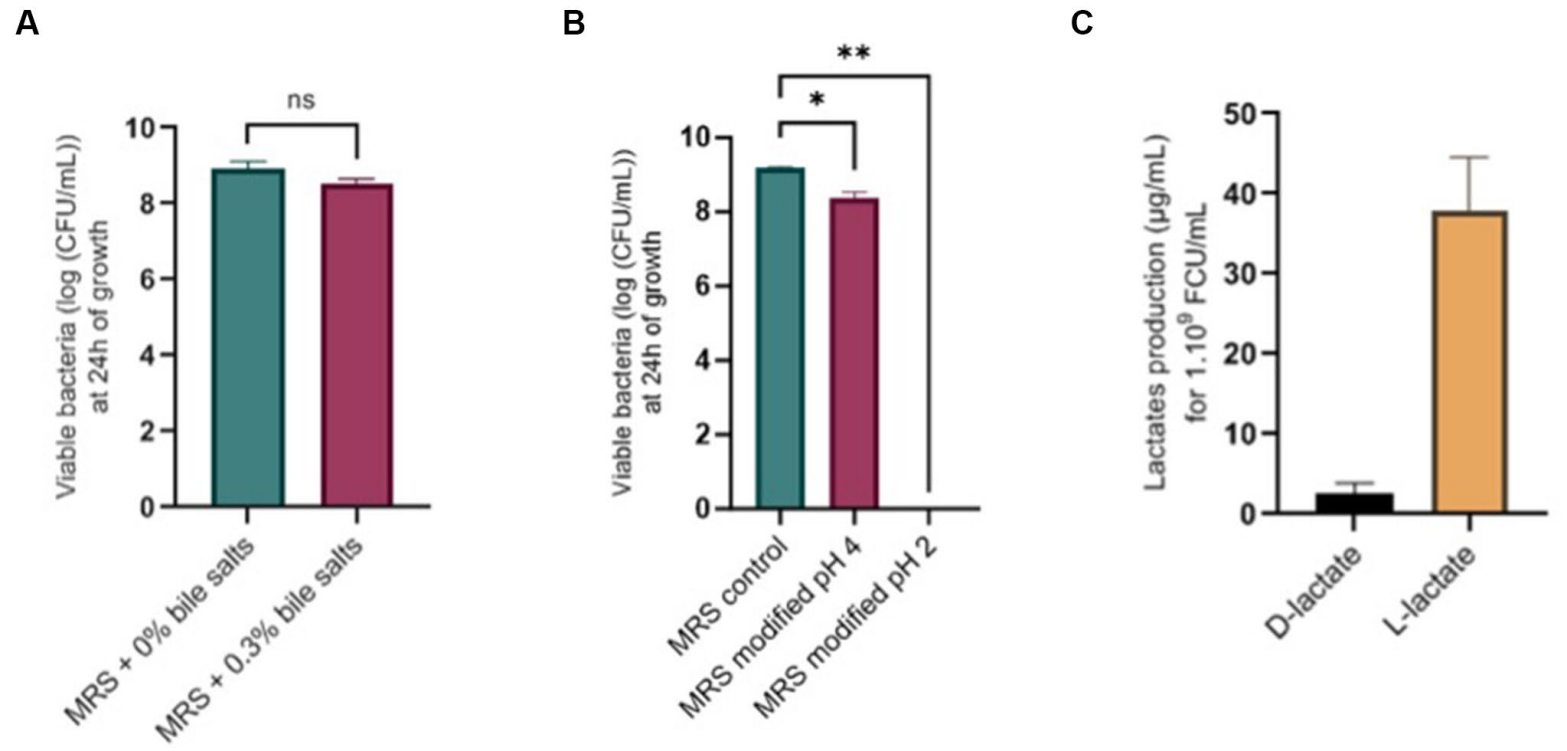
Physiological potentialities of *L. salivarius* CNCM I-4866. **(A)** Survival of *L. salivarius* CNCM I-4866 after 24-h growth in media with 0.3% or 0% bile salts, expressed in log of CFU/ml; **(B)** Survival of *L. salivarius* CNCM I-4866 after 24-h growth in media at pH 4 or pH 2, expressed in log of CFU/ml; **(C)** D-lactate and L-lactate production of *L. salivarius* CNCM I-4866. Results of Mann–Whitney *U*-tests: *p < 0.05, **p < 0.01, ***p < 0.001, and ****p < 0.0001.

No significant difference was found after growth in media with 0.3% bile salts (Figure 8A) or after 1 h of exposition to 0.3% bile salts. Concerning tolerance to acidic conditions, *L. salivarius* CNCM I-4866 growth was found to be slightly impacted by pH 4 and inhibited at pH 2 (Figure 8B). Moreover, no hemolytic activity was found after the growth of *L. salivarius* CNCM I-4866 on blood agar.

### *Ligilactobacillus salivarius* CNCM I-4866 produced mainly L-lactate as a fermentation product

We have measured D-lactate and L-lactate production from the strain *L. salivarius* CNCM I-4866 which produced mostly L-lactate (Figure 8C).

### *Ligilactobacillus salivarius* CNCM I-4866 is a potential producer of exopolysaccharides

**Figure 9:**
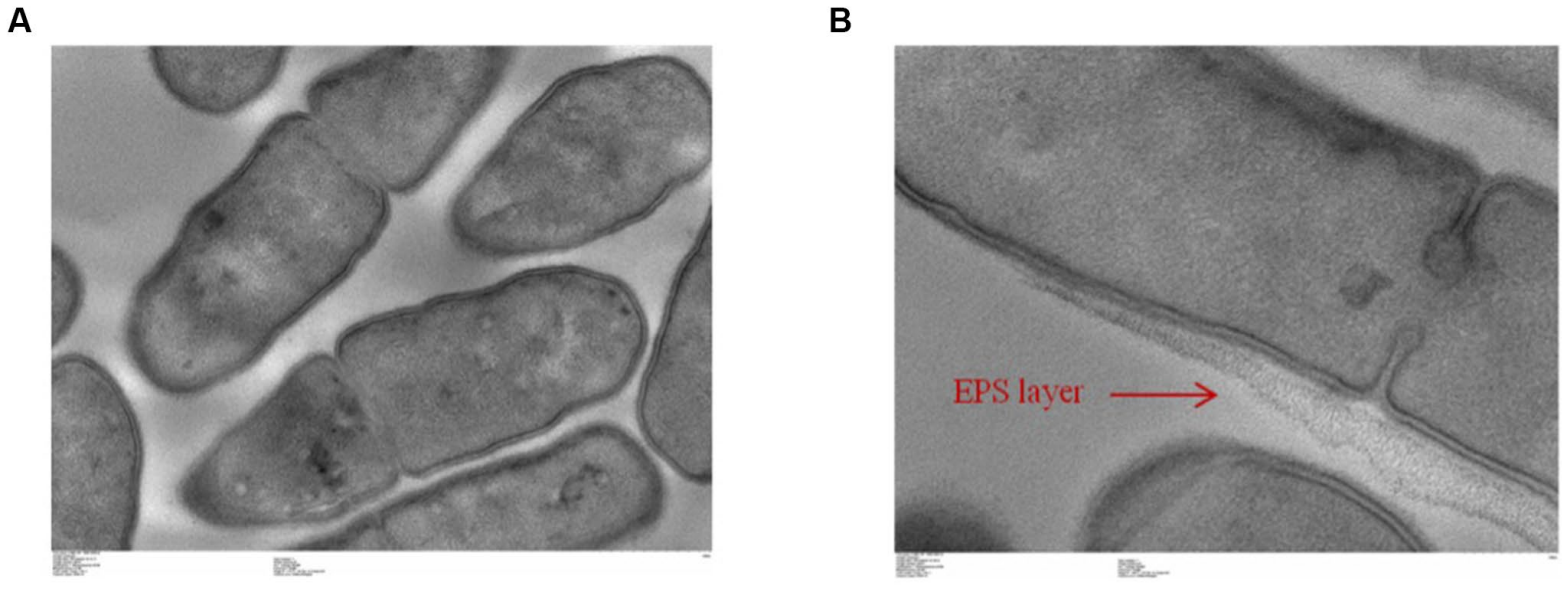
Transmission electronic microscopic visualization of *L. salivarius* CNCM I-4866 **(A,B)**.

Finally, potential exopolysaccharide production was first assessed by the Ropy test. The Ropy test was positive (data not shown). This test was completed with a global analysis of the structure of *L. salivarius* CNCM I-4866 by transmission electron microscopy (Figures 9A,B). This result has shown a structure that is compatible with an EPS layer, suggesting EPS production by the strain.

### Antibiotic resistance analysis of *Ligilactobacillus salivarius* CNCM I-4866 strain *in vitro* and *in silico*

To ensure the health safety of *L. salivarius* CNCM I-4866, phenotypic resistance to several antibiotics was determined according to the EFSA recommendations (Table 1). The results showed that the strain is sensitive to ampicillin, gentamicin, streptomycin, erythromycin, clindamycin, tetracycline, and chloramphenicol and resistant to kanamycin.

**Table 1:** Phenotypic antibiotic resistance according to the EFSA recommendations for *L. salivarius* CNCM I-4866.

**Antibiotic**	**MIC values (mg/L)**	**Cutoff values EFSA (mg/L)**
Ampicillin	0.25–0.38	4
Gentamicin	4	16
Kanamycin	>256	64
Streptomycin	48	64
Erythromycin	0.5	1
Clindamycin	0.125	1
Tetracycline	0.75–1	8
Chloramphenicol	2	4

Minimal inhibitory concentration (MIC) values are compared with the cutoff values from the EFSA guidelines.

Furthermore, the *L. salivarius* CNCM I-4866 genome was sequenced by whole-genome sequencing. Potential genes for antibiotic resistance were searched in online databases. No gene responsible for antibiotic resistance was found according to our thresholds.

### Analysis of the presence of bacteriophage and bacteriocin *in vitro* and *in silico*

Potential phage or bacteriocin production has been determined *in silico* and *in vitro. In silico* Phaster analysis highlighted the presence of an intact prophage, B025, a *Listeria* prophage (score of 120, length of 43.7 Kb, and region from 121,049 to 164,766 with a percentage of GC of 33.63%). Induction assay and research on lytic plaques with a sensitive strain for this phage have shown that this prophage was not active in our conditions (data not shown). Moreover, *in silico*, the bacteriocin enterolysin was found to be potentially active. After testing on a sensitive strain, no halo was observed, indicating that the bacteriocin was not active.

## Discussion

In Western countries, IBDs are widespread chronic diseases, with no curative treatment available for the moment (Aldars-Garcia et al., 2021). Therapies based on supplementation with beneficial microorganisms have been pointed out as a potential co-treatment in the management of the symptoms. This approach could be performed with traditional probiotics such as lactic acid bacteria (Doron et al., 2005; Cheng et al., 2020). In this study, we characterized a Lactobacillaceae strain, *L. salivarius* CNCM I-4866, as a potential probiotic strain to manage and moderate intestinal inflammation.

First, the anti-inflammatory properties of our strain were assessed in a DNBS-induced colitis model. This acute inflammation displays many related features of Crohn’s disease, making it an approaching model for this pathology (Wallace et al., 1995). After treatment with CNCM I-4866, colon macroscopic scores improved significantly compared with DNBS control. These results were in accordance with microscopic scores, including damage to colonic epithelial structure and immune cell infiltration, which *L. salivarius* CNCM I-4866 tended to improve. To assess the immunomodulatory effects of the strain in our DNBS model, colonic MPO, an enzyme found in the intracellular granules of neutrophils, and serum LCN-2, a non-invasive marker of inflammation, were quantified. Administration of *L. salivarius* CNCM I-4866 tended to decrease both markers when compared with the DNBS control. Colonic inflammation has been proven to be linked with a dysfunction of intestinal barrier function and, therefore, an increase in permeability as it is observed in IBD patients (Michielan and D’Inca, 2015; Salvo-Romero et al., 2015). By determining sCD14 levels in serum, we have observed that *L. salivarius* CNCM I-4866 tends to maintain intestinal barrier integrity in cases of inflammation. This model is a robust model for probiotic identification as it has employed other bacteria with well-known probiotic capacities, such as *Faecalibacterium prausnitzii* (Martin et al., 2014) or several *Lactobacillus* strains (Benitez-Cabello et al., 2020).

Additionally, colonic transcriptome analysis by comparing the DNBS-Vehicle group with the DNBS-*L. salivarius* CNCM I-4866-treated group has revealed that genes implied in inflammation and, more precisely, pro-inflammatory cytokine production were less expressed when mice were treated with CNCM I-4866. Conversely, based on the z-score, the expression of the anti-inflammatory cytokine IL-10 was higher in the treated group. The major pathway upregulated in DNBS-Vehicle is the tumor environment pathway, which includes not only cancer cells but also many immune cells that occur during inflammation development. Thereby, IL-1β is a major inflammatory cytokine that mediates other pro-inflammatory cytokines, such as TNF-α or IL-12 (Wang et al., 2017). As these cytokines were upregulated in the control DNBS group (DNBS-Vehicle) compared with the DNBS-CNCM I-4866 group, we can assume that our probiotic candidate has the ability to pacify gene expression linked to immune response. A previous study has shown an equivalent pattern of immunity modulation in a DSS-induced colitis model with a *Lactobacillus plantarum* strain (Wu et al., 2022). To summarize, *L. salivarius* CNCM I-4866 seems to alleviate inflammatory bursting caused by DNBS colitis.

Taken together, these *in vivo* findings indicate that the *L. salivarius* CNCM I-4866 strain could be a good probiotic candidate to manage and reduce intestinal inflammation found in IBD patients. To go further on the understanding of the beneficial properties of CNCM I-4866 and, more specifically, immunomodulatory effects, two cellular models were used: TNF-α-activated HT-29 cells and PBMCs. Consequently, the anti-inflammatory effect was evaluated by measuring pro-inflammatory cytokine (IL-8) concentration in a co-incubation model of HT-29 cells with *L. salivarius* CNCM I-4866 bacteria after the TNF-α challenge. IL-8 secretion was shown to be increased in IBD patients and correlated with mucosal inflammation (Daig et al., 1996). De Oliveira et al. (2013) described that this interleukin is responsible for neutrophil activation in the case of inflammation. Previous studies have already evaluated probiotic aptitude with this parameter (Martin et al., 2019; Maillard et al., 2023). Our strain has shown a good ability to reduce IL-8 production as compared with inflamed cells alone, confirming the anti-inflammatory properties observed *in vivo.*

To have a better picture of the impact of *L. salivarius* CNCM I-4866 on immunity at the peripheral level, we have determined its capacity to regulate the production of IL-10, IL-12, and TNF-α on human PBMC cells. High secretion of IL-10 was measured with CNCM I-4866 by comparing it with controls. Rogler and Andus (1998) have described the importance of this cytokine in immune homeostasis in IBD patients as an anti-inflammatory response. On the other hand, the pro-inflammatory cytokine IL-12 leads to a Th1 immune-mediated response with the differentiation of T-helper cells. The IL-10/IL-12 ratio is described as a reliable indicator for establishing the inflammatory profile of a strain, with a high ratio associated with an anti-inflammatory profile (Foligne et al., 2007). By comparing it with the well-known probiotic *L. rhamnosus* GG, CNCM I-4866 produced significantly more IL-10 and less IL-12, revealing a high IL-10/IL-12 ratio. These results are in accordance with those of Foligne et al. (2007), who have shown that *L. salivarius* Ls33 has a pronounced anti-inflammatory profile based on the IL-10/IL-12 ratio. The low TNF-α/IL-10 ratio, compared with *L. rhamnosus* GG, allowed us to confirm this anti-inflammatory pattern. These outcomes, taken with *in vivo* and IL-8 results, point out a strong anti-inflammatory profile of *L. salivarius* CNCM I-4866.

As mentioned above, intestinal permeability is impacted in the case of inflammation and, therefore, in IBD pathologies. In the DNBS model, CNCM I-4866 tends to maintain barrier permeability by using the sCD14 marker. Furthermore, we have evaluated this capacity in an *in vitro* model. Caco-2 cells are exposed to TNF-α, which disrupts tight junctions and increases epithelial barrier permeability. *L. salivarius* CNCM I-4866 treatment was able to restore barrier integrity in Caco-2 cells challenged with TNF-α. A study has shown that *L. rhamnosus* GG could attenuate permeability dysfunction induced by TNF-α and IFNγ by inhibiting the NF-κB pathway (Donato et al., 2010). *Lactobacillus plantarum* MB452 was also found to enhance intestinal barrier function by modulating tight junction proteins (Anderson et al., 2010). For CNCM I-4866, the *in vivo* effect on permeability was not pronounced, but *in vitro* assay with TEER highlighted the beneficial property of maintaining the permeability of our strain. Additional experiments should be carried out for a better understanding of the partial transferability of these *in vitro* results to the *in vivo* preclinical context and to further analyze the underlying mechanisms.

Rossi et al. (2015) and Hidalgo-Cantabrana et al. (2016) have shown that EPS production by probiotics candidates was a key parameter for them to exert their anti-inflammatory properties. To continue with a deeper characterization of *L. salivarius* CNCM I-4866, we have thus determined EPS production. EPS secretion is known to be a criterion for an adequate probiotic candidate as it has health benefits (Juraskova et al., 2022). With electronic transmission microscopy, we have observed that our strain possesses a potential EPS. This observation is supported by the Ropy test, indicating that CNCM I-4866 produces Ropy-linked EPS. Several mechanisms are known to be implied in beneficial effects exerted by EPS, for example modulation of intestinal microbiota (Salazar et al., 2008).

Even if the underlying mechanisms are not well known, it is known that IBDs are linked to a microbiota imbalance between commensal and pathogenic bacteria. Indeed, some pathogen populations are increased in the case of IBD, such as *Salmonella, Escherichia coli,* or *Listeria monocytogenes* (Axelrad et al., 2021). As *L. salivarius* species are well known to exert antimicrobial activity (Tinrat et al., 2011; Messaoudi et al., 2013), we have evaluated this capacity for CNCM I-4866 against eight intestinal pathogens. *L. salivarius* CNCM I-4866 was able to inhibit two *Salmonella* strains: one *E. coli* strain and one *S. aureus* strain. This property, potentially due to lactic acid production, is an interesting feature that can be considered for further applications. In a previous study, Kang et al. (2017) highlighted the anti-microbial mechanisms of *L. salivarius* strains against *S. aureus*, such as the secretion of anti-staphylococcal proteins.

Beyond its health-beneficial properties, we wanted to ensure that *L. salivarius* CNCM I-4866 was a good probiotic candidate (European Food Safety Authority, 2018). As a potential human probiotic, tolerance to bile salts is essential, as it will allow the bacteria to reach the lower intestinal tract. Thereby, we have shown that *L. salivarius* CNCM I-4866 could resist 0.3% bile salts, corresponding to the physiological concentration in the human gastrointestinal tract (Chateau et al., 1994; Prete et al., 2020). This capacity constitutes an advantage for the *in vivo* survival of the strain. In previous studies, probiotic candidate strains were screened on this parameter, and *Lactobacillus* strains have also shown good survival at 0.3% bile salts (Khiralla et al., 2015). The ability to tolerate bile salts is commonly known due to bile salt hydrolase activities (Noriega et al., 2006). However, Pan et al. (2021) have described the fact that, for some *L. salivarius* strains, other mechanisms could be responsible for this property. The growth of our strain was impacted by the low pH (pH 4 and pH 2) that mimic the passage in the gastrointestinal tract and, more specifically, gastric conditions. However, probiotic strains are often administered orally in a protective vehicle that ensures the viability of strain during its passage through the gastrointestinal tract (Tee et al., 2014).

Adhesion to the intestinal mucosa constitutes a key parameter in selecting a probiotic, as it allows the strain to persist and exert its health-beneficial effects for a longer period. *L. salivarius* CNCM I-4866 possessed good adhesion capacities on several cell lines and mucus at a similar or even better degree than the well-known probiotic, *L. rhamnosus* GG. Adhesion mechanisms are well described and can be either specific to adhesion proteins (fibronectin, collagen, mucin, and laminin) or unspecific to binding to hydrophobic surfaces (de Wouters et al., 2015). Additionally, CbpA protein was identified in *L. salivarius* REN as essential for its adhesion to the HT-29 line (Wang et al., 2017). For *L. rhamnosus* GG, functional analysis has revealed that SpaCBA pili act as an essential factor in adhesion and immunomodulation (Lebeer et al., 2012), as is the case of SpaFED pili for *L. rhamnosus* CNCM-I3690 (Martin et al., 2023).

Regarding safety concerns, antibiotic resistance constitutes a major issue nowadays, and the risk of resistance gene dissemination should be limited to its maximum (Li et al., 2020). Following the EFSA recommendations (European Food Safety Authority, 2021), no antibiotic-resistance gene was found in our strain. Nevertheless, phenotypic antibiotic resistance has highlighted the resistance to kanamycin. It is well described that *Lactobacillus* are frequently resistant to kanamycin due to intrinsic resistance (Anisimova and Yarullina, 2019; Campedelli et al., 2019). As no gene is detected, *L. salivarius* CNCM I-4866 is validated on safety aspects. Additionally, no hemolytic activity was found for our strain. Lactic acid bacteria, as their name suggests, are high producers of lactate. Iraporda et al. (2016) have established that L-lactate treatment could alleviate intestinal inflammation in a mouse TNBS model. However, it has been shown that D-lactate accumulation can lead to acidosis in people with short bowel syndrome (Mack, 2004). As *L. salivarius* CNCM I-4866 produces mostly L-lactate and very little D-lactate, this strain is suitable for these patients.

In conclusion, our study has shown that a new strain, *L. salivarius* CNCM I-4866, displays strong anti-inflammatory capacities *in vitro* and *in vivo*. Even if further research could be useful to better understand the mechanisms involved or to test this strain on moderate inflammation, CNCM I-4866 is confirmed to be a promising probiotic candidate to alleviate inflammation at the preclinical level on a DNBS model, mimicking IBD and, more specifically, Crohn’s disease. Nevertheless, a human clinical trial should be performed to confirm its potential.

## Data availability statement

The data presented in the study are deposited in the biosamplehelp@ncbi.nlm.nih.gov repository, accession number SAMN37542358.

## Ethics statement

Ethical approval was not required for the studies on humans in accordance with the local legislation and institutional requirements because only commercially available established cell lines were used. The animal study was approved by Experiments were performed in accordance with European Union legislation on animal welfare and were approved by COMETHEA, our local committee on animal experimentation (n° 16744–201807061805486) and in compliance with the ARRIVE relevant guidelines. The study was conducted in accordance with the local legislation and institutional requirements.

## Author contributions

CC: Conceptualization, Formal analysis, Investigation, Methodology, Validation, Writing – original draft. SC: Investigation, Validation, Methodology, Writing – review & editing. CK: Investigation, Validation, Methodology, Writing – review & editing. LM: Investigation, Validation, Methodology, Writing – review & editing. FC: Investigation, Validation, Methodology, Writing – review & editing. PL: Conceptualization, Funding acquisition, Validation, Writing – review & editing. RM: Conceptualization, Funding acquisition, Investigation, Methodology, Project administration, Supervision, Validation, Writing – review & editing.

## Footnotes

^1^ https://card.mcmaster.ca/home

^2^ https://cge.food.dtu.dk/services/ResFinder/

^3^ https://phaster.ca/

^4^ http://bagel4.molgenrug.nl/
